# Jolkinolide B induces cell cycle arrest and apoptosis in MKN45 gastric cancer cells and inhibits xenograft tumor growth *in vivo*

**DOI:** 10.1042/BSR20220341

**Published:** 2022-06-27

**Authors:** Hao Zhang, Jiayi Qian, Ming Jin, Li Fan, SongJie Fan, Hong Pan, Yang Li, Ningning Wang, Baiyu Jian

**Affiliations:** 1 Research Institute of Medicine and Pharmacy, Qiqihar Medical University, Qiqihar 161000, P. R. China; 2 College of Pharmacy, Qiqihar Medical University, Qiqihar 161000, P. R. China

**Keywords:** Apoptosis, Cell Cycle, Jolkinolide B

## Abstract

Gastric cancer is one of the most common digestive carcinomas throughout the world and represents high mortality. There is an urgent quest for seeking a novel and efficient antigastric cancer drug. *Euphorbia fischeriana* Steud had long been used as a traditional Chinese medicine for the treatment of cancer. According to the basic theory of traditional Chinese medicine, its antitumor mechanism is ‘to combat poison with poison’. However, its effective material foundation of it is still ambiguous. In our previous work, we studied the chemical constituents of *E. fischeriana* Steud. Jolkinolide B (JB) is an ent-abietane-type diterpenoid we isolated from it. The purpose of the present study was to investigate the antigastric effect and mechanism of JB. Results revealed that JB could suppress the proliferation of MKN45 cells *in vitro* and inhibit MKN45 xenograft tumor growth in nude mice *in vivo*. We further investigated its anticancer mechanism. On the one hand, JB caused DNA damage in gastric cancer MKN45 cells and induced the S cycle arrest by activating the ATR-CHK1-CDC25A-Cdk2 signaling pathway, On the other hand, JB induced MKN45 cells apoptosis through the mitochondrial pathway, and ultimately effectively inhibited the growth of gastric cancer cells. These results suggest that JB appears to be a promising candidate drug with antigastric cancer activity and warrants further research.

## Introduction

Gastric cancer is the most common malignant tumor of the digestive system [1]. With the changes in economic situation and lifestyles, the morbidity of gastric cancer increases gradually [2]. Although the level of comprehensive treatment for gastric cancer continues to improve, the 5-year survival rate of gastric cancer patients is still lower than 40%, and there is a lack of effective treatments in clinics [3]. Therefore, seeking novel and efficient antigastric cancer drugs becomes a hot point for pharmaceutical researchers.

Natural products are a vital source for drug discovery [4]. *Euphorbia fischeriana* Steud is used as a traditional Chinese medicine to treat edema, ascites, and cancer [5,6]. Among them, its anticancer activity has attracted the most attention from researchers recently. It is reported that *E. fischeriana* Steud contains abundant diterpenoids [7,8]. Diterpenoids are the hotspot of drug research and development due to their wide structural diversity and various biological activities. Many diterpenoids have been reported to have anticancer activity, such as paclitaxel, and triptolide [9,10]. This information prompted us to further investigate the anticancer effect of diterpenoids isolated from *E. fischeriana* Steud.

Our research team has conducted relevant research on the chemical composition and pharmacological properties of *E. fischeriana* Steud. Jolkinolide B (JB) is an ent-abietane-type diterpenoid isolated from petroleum ether extract of *E. fischeriana* Steud. Our previous study has shown that JB could inhibit the growth of breast and lung cancer cells. However, its role in the regulation of gastric cancer remains elusive [11,12]. In the current study, we aim to investigate its antigastric cancer effect *in vivo* and *in vitro* and explore its antigastric cancer mechanism from the perspectives of cell cycle and cell apoptosis, which can enrich the scientific connotation of treating gastric cancer by *E. fischeriana* Steud, lay the foundation for further drug development of JB, and provide a new clue for gastric cancer treatment.

## Materials and methods

### Cell line and reagents

Human low-differentiated gastric cancer MKN45 cell line was purchased from the Cell Resource Center (IBMS, CAMS/PUMC). Cells were grown in RPMI 1640 medium (GIBCO, Grand, NY, U.S.A.) containing 10% fetal bovine serum in a humidified incubator at 37°C, 5% CO_2_, and digested with 0.25% trypsin. Compound JB was obtained from Professor Li-Na Guo (Institute of Traditional Chinese Medicine and Natural Products, Qiqihar Medical University).

### Cell viability assay

MKN45 cells were inoculated into 96-well plates and treated with different concentrations of JB (0, 5, 10, 20, 40, and 80 µM) for 24, 48, and 72 h, respectively. Then, MTT solution was added to plates and incubated for 4 h. Later, the medium was discarded and DMSO was added to solubilize the crystals. The absorbance was detected on a microplate reader at 492 nm. Then, the cell viability and 50% inhibitive concentration (IC50) of JB was calculated.

### Colony survival assay

Cell proliferation was assessed with clone formation assay. Briefly, MKN45 cells were plated onto 6-well plates and treated with JB with concentration gradients (0, 10, 20, 40 μM) for 24 h. The cells were collected, re-suspended, counted, and seeded in 6-well plates (500 cells per well). Cells were fixed with 100% methanol and stained with 0.5% crystal violet after incubation for 2 weeks. Then, colonies were counted and photographed.

### EdU proliferation assay

MKN45 cells were seeded onto 6-well plates and treated with the indicated agents. The cell proliferation was measured by BeyoClick™ EdU Cell Proliferation Kit with Alexa Fluor 488 (Beyotime Biotechnology, China). The operations were conducted according to the manufacturers’ protocol. Then, EdU-positive staining rate was analyzed via flow cytometry (BD Biosciences).

### Cell cycle analysis

The role of JB in cell cycle distribution was detected by flow cytometry with propidium iodide (PI) staining (Beyotime Biotechnology, China). After drug treatment, cells were collected and fixed in 70% ethanol at 4°C overnight. Next, cells were washed twice with cold PBS and subsequently labeled with PI at 37°C for 30 min in dark. After that, the stained cells were measured by flow cytometry.

### Immunostaining analysis

For immunostaining, MKN45 cells were grown on coverslips and treated with the indicated agents. MKN45 cells were fixed with 4% paraformaldehyde for 30 min and permeabilized with 0.2% Triton X-100 for 15 min. Samples were subsequently blocked in 5% bovine serum albumin (BSA) in PBS for 30 min at room temperature, followed by incubating with antibodies against γ-H2AX (Cell Signaling Technology, U.S.A.) at 4°C overnight. The cells were washed three times with 1×TBST and incubated with Alexa Fluor 594 Conjugate antirabbit antibody (Absin, China) for 1 h and counterstained with DAPI. Immunofluorescence images were acquired under Zeiss LSM confocal microscope.

### AO/EB dual staining

Cell morphology of apoptosis was determined by the Acridine orange/Ethidium bromide (AO/EB) fluorescence staining. MKN45 cells were seeded into 6-well plates and treated with various concentrations of JB. Then, the media were then removed and the cells were washed with PBS twice and subsequently stained with the AO/EB mixture for 10 min at room temperature (in dark). At last, the stained cells were visualized by a confocal fluorescence microscope.

### Transmission electron microscope

MKN45 cells were inoculated into 6-well plates and treated with selected concentrations of JB. The cells were harvested, fixed in 2.5% glutaraldehyde, and dyed with osmic acid. Afterward, the samples were dehydrated with graded acetone series (50, 70, 90, 100%) and embedded in epoxy-resin blocks. Then, ultrathin sections (100 nm) were made and stained with uranium acetate-lead citrate. Finally, cells were observed by a transmission electron microscope (TEM, HITACHI, Japan)

### Annexin-V-FITC/PI staining assay

Cell apoptosis was analyzed by Annexin V-FITC/PI detection kit (Beyotime Biotechnology, China). In brief, MKN45 cells were seeded into 6-well plates and various concentrations of JB, digested with pancreatin, and washed twice with PBS. Subsequently, cells were incubated with Annexin V-FITC and PI in the dark at room temperature for 20 min. The fluorescence intensity of the stained cells was measured by a flow cytometer.

### Mitochondrial membrane potential assay

Mitochondrial membrane potential (ΔΨm) of MKN45 cells was detected through staining assay following the kit instructions (Beyotime, Hangzhou, China). The MKN45 cells treated by JB were collected and stained with JC-1 probes for 30 min at 37°C in darkness. After incubation, cells were washed with JC-1 staining buffer and then ΔΨm was measured by flow cytometry.

### Western blot analysis

The MKN45 cells treated by JB were lysed in RIPA buffer with phosphatase inhibitor. Protein concentrations were measured by BCA Protein Assay Kit (Biyuntian, China). Total protein extracts were separated by 8–12% SDS-PAGE, transferred to PVDF membranes (Millipore Corp, Bedford, U.S.A.), and blocked with 5% BSA for 2 h at room temperature. After sealing the PVDF membrane, the primary antibodies against ATR, p-ATR, CHK1, p-CHK1, CDC25A, CDK2, Cyclin A, γ-H2AX, Cytochrome c, Cleaved Caspase-3, Cleaved Caspase-9, BAX, BCL-2, and β-Actin (Cell Signaling, U.S.A.) were added and incubated overnight at 4°C. Next, membranes were washed three times with TBS—Tween-20 and subsequently with secondary antibody (Cell Signaling Technology, U.S.A.) for 1 h at room temperature. Then, membranes were washed three times again. Finally, an ECL chromogenic kit was used to visualize the bands (Thermo Scientific, Rockford, IL, U.S.A.). Antibodies and concentrations used in the present study can be found in Supplementary Table S1.

### *In vivo* antitumor activity

All animal experiments took place in SPF Animal Laboratory of Qiqihar Medical University. Ethical approval has been sought from the Ethics Committee of Qiqihar Medical University (No. QWU-AECC-2020-60). Balb/c nude mice (4–5 weeks of age) were purchased from Liaoning Changsheng biotechnology (Liaoning, China). MKN45 cells were implanted into the armpit of nude mice. Forty nude mice were randomly divided into four groups of ten mice in each group. The mice were treated with JB at the doses of 10, 20, and 40 mg/kg by intragastric administration (i.g.) daily for 2 weeks, respectively. Control mice were given an equal volume of normal saline. The body weight and two perpendicular tumor diameters (a and b) were measured every 2 days, and the tumor volume (V) was calculated according to the following formula: V = ab2π/6, where a is the longest diameter, and b is the shortest diameter, during the experiment process. At the end of the experiment, nude mice were anesthetized with pentobarbital (60 mg/kg) and killed by cervical dislocation. Then, tumor tissues were taken out and weighed.

### Statistical analyses

Statistical analysis was carried out with SPSS Statistics 24.0. Data derived from at least three independent experiments were presented as mean ± standard deviation (SD). Multiple-group comparisons were performed using a one-way analysis of variance (ANOVA). All statistical tests were performed with the bilateral test, *P<0.05 was considered statistically significant, and **P<0.01 was considered highly statistically significant.

## Results

### JB inhibited the proliferation of MKN45 cells

**Figure 1:**
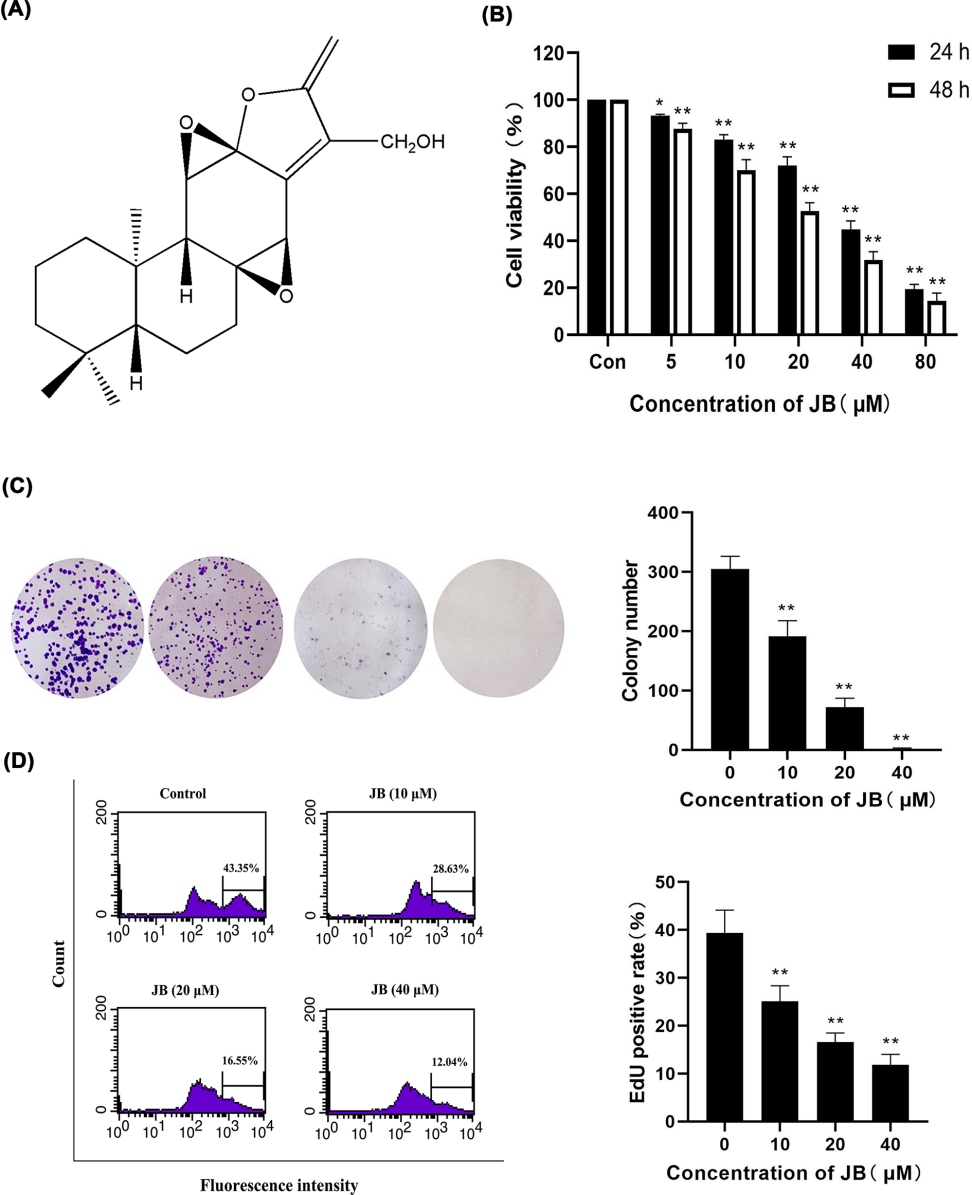
JB inhibited the growth of MKN45 cells (**A**) Chemical structure of JB. (**B**) MKN45 cells were treated with the indicated concentrations of JB for 24 and 48 h, followed by cell viability assessment using MTT assay. (**C**) MKN45 cells were treated with JB 10, 20, 40 μM for 24 h, and clonal formation rate was detected by colony survival assay. (**D**) MKN45 cells were treated with JB 10, 20, 40 μM for 24 h, and DNA synthesis was analyzed by EdU incorporation experiment. All data are expressed as means ± SD of three independent experiments. *P<0.05, **P<0.01 compared with the control group.

The chemical structure of JB is shown in Figure 1A. We investigated the growth inhibition of JB in MKN45 cells using an MTT assay. JB significantly suppressed MKN45 cell survival in a time- and dose-dependent manner (Figure 1B). Half-maximal IC50 value of JB to MKN45 cells for 24 and 48 h were (44.69±2.26) and (33.64±3.64) μM, respectively. Based on the results, the effective drug concentrations were chosen for the follow-up experiments. The clone formation assay indicated that JB inhibited the clone formation capacity in MKN45 cells (Figure 1C). In addition, JB can also inhibit EdU incorporation during DNA synthesis. EdU assay demonstrated that JB markedly decreased the number of EdU-positive MKN45 cells in a dose-dependent when compared with the control group (Figure 1D). These findings indicated that JB inhibited the proliferation of MKN45 cells.

### JB induced cell cycle arrest of MKN45 cells

**Figure 2:**
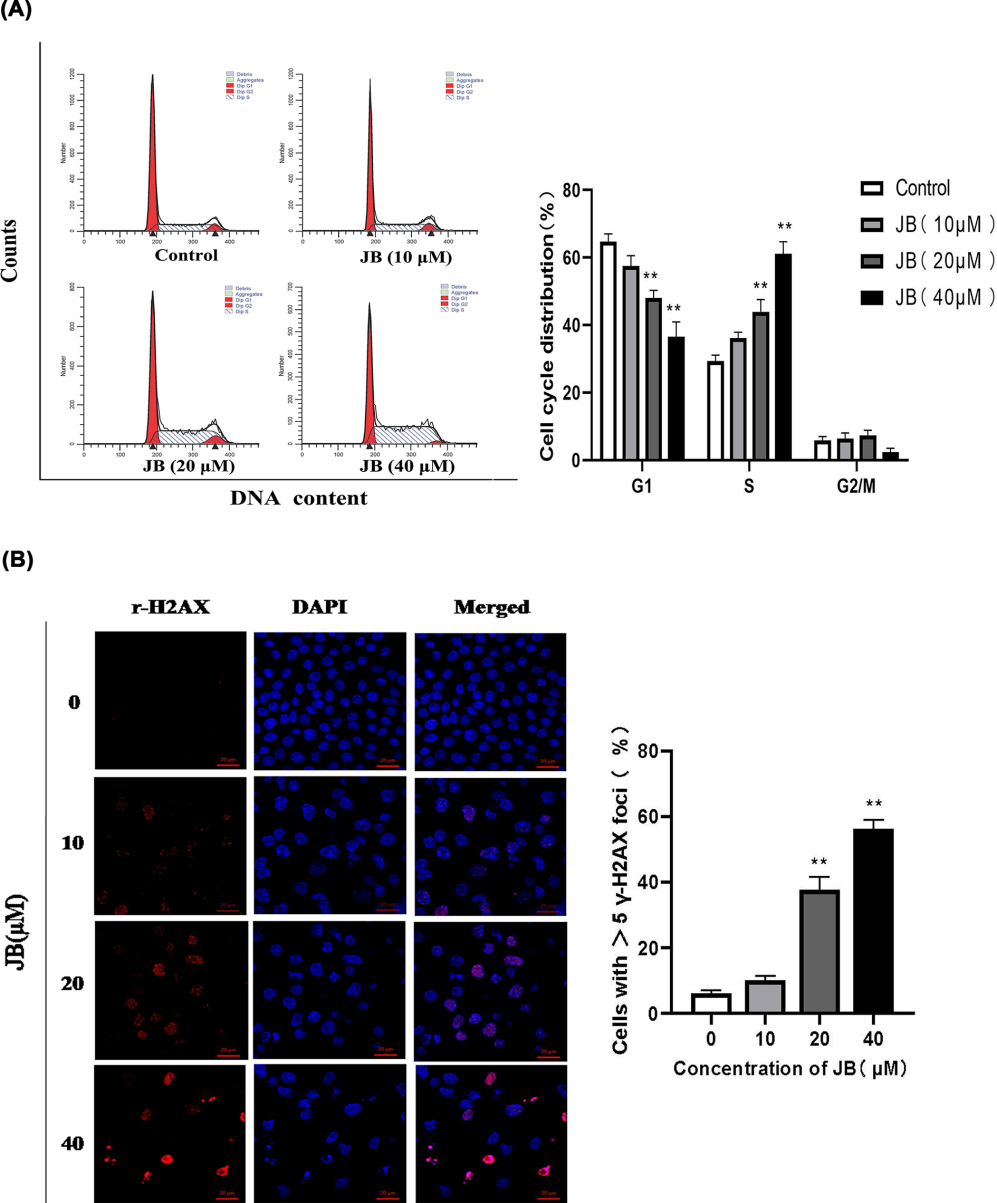
JB induced DNA damage and S cycle arrest in MKN45 cells The cells were incubated with or without various concentrations of JB for 24 h. (**A**) The cell cycle distribution was analyzed by flow cytometry after PI staining. (**B**) γ-H2AX foci were assessed by confocal microscopy. Data are expressed as means ± SD of three independent experiments. *P<0.05, **P<0.01 compared with the control group.

**Figure 3:**
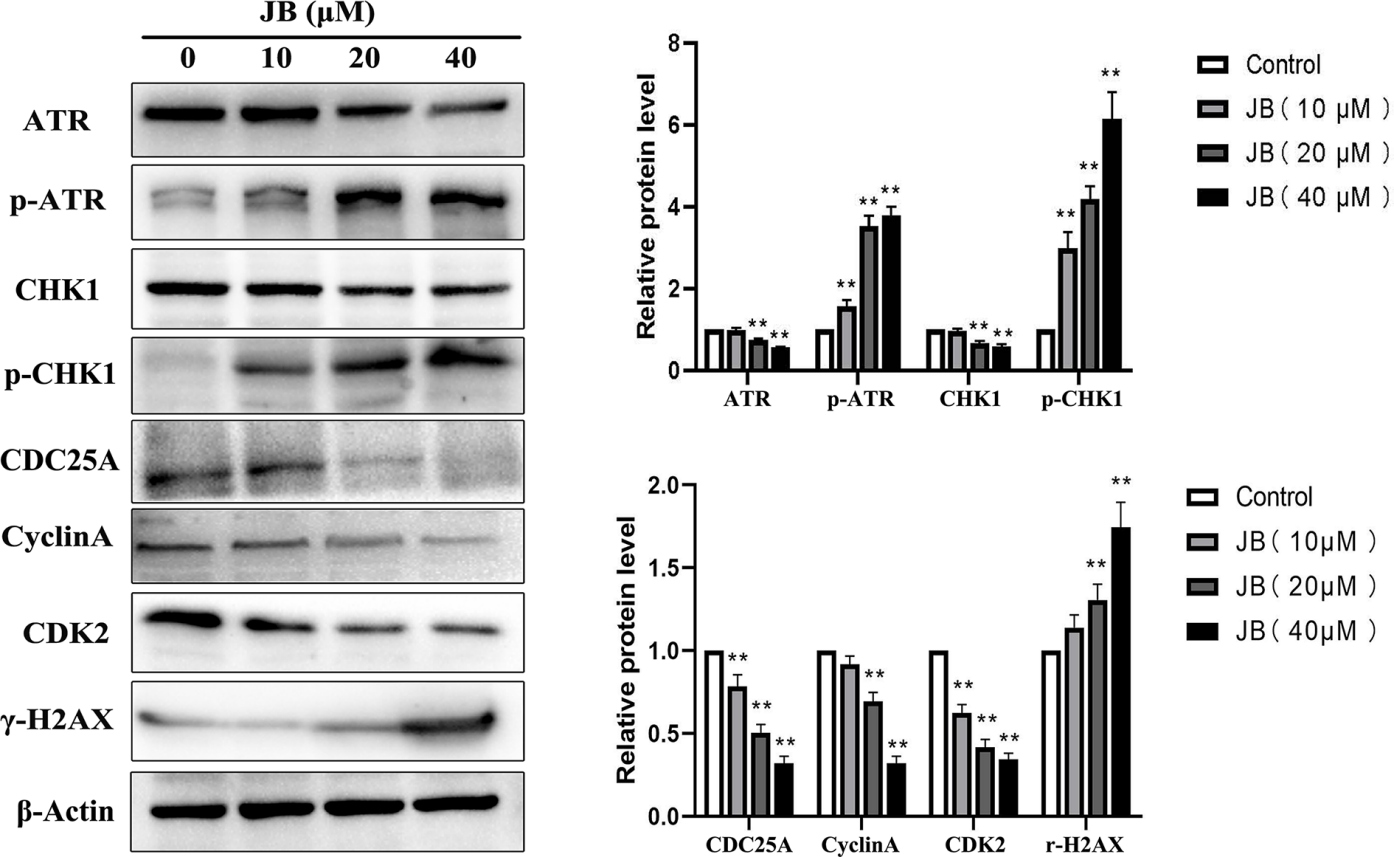
JB activated the ATR-CHK1 signal pathway in MKN45 cells The cells were incubated with or without various concentrations of JB for 24 h. The protein expression levels of ATR, p-ATR, CHK1, p-CHK1, CDC25A, CyclinA, CDK2, and r-H2AX were detected by western blotting. Data are expressed as means ± SD of three independent experiments. *P<0.05, **P<0.01 compared with the control group.

As cell proliferation is regulated by the cell cycle, we deduced that the antiproliferative effect of JB is associated with cell cycle alterations. We then detected the effect of JB on cell cycle distribution in MKN45 cells via flow cytometry. The result showed that JB significantly increased the percentage of cells in S phase in a concentration-dependent manner (Figure 2A). Regarding cell cycle markers, we further evaluated the expression of regulatory molecules associated with the S phase. We found that JB could down-regulate Cyclin A, CDC25A, and CDK2 expressions in a dose-dependent manner (Figure 3 and Supplementary Figure S1). Our studies above have shown that JB induced S arrest in MKN45 cells.

### JB induced MKN45 cells DNA damage and ATR-CHK1 signal pathway activation

Studies have revealed that many anticancer drugs induce S-phase cell cycle arrest of tumor cells by inducing cell DNA damage and activating of ATR-CHK1 signal pathway. We speculated that JB might exert its anticancer effects via inducing DNA damage and activating the ATR-CHK1 signal pathway in MKN45 cells. γ-H2AX is currently recognized as a molecular marker of DNA damage and DNA double-strand breaks (DSB). In order to evaluate the effect of JB on DNA, we performed immunostaining and western blot to measure the expression of γ-H2AX. As shown in Figures 2B and 3, protein levels of γ-H2AX were up-regulated in a dose-dependent manner, which suggested JB-induced DSB in MKN45 cells. To investigate whether the ATR-CHK1 signaling pathway is a relevant molecular mechanism in JB-induced S-phase cell cycle arrest. We measured the expression of key proteins related to this signal pathway via western blot in MKN45 cells. The result showed that JB induced phosphorylation of ATR and CHK1 (Figure 3 and Supplementary Figure S1). These results suggested that the ATR-CHK1 pathway may play an important role in JB-induced cell cycle arrest in MKN45 cells.

### JB induced apoptosis in MKN45 cells through the mitochondrial apoptotic pathway

**Figure 4:**
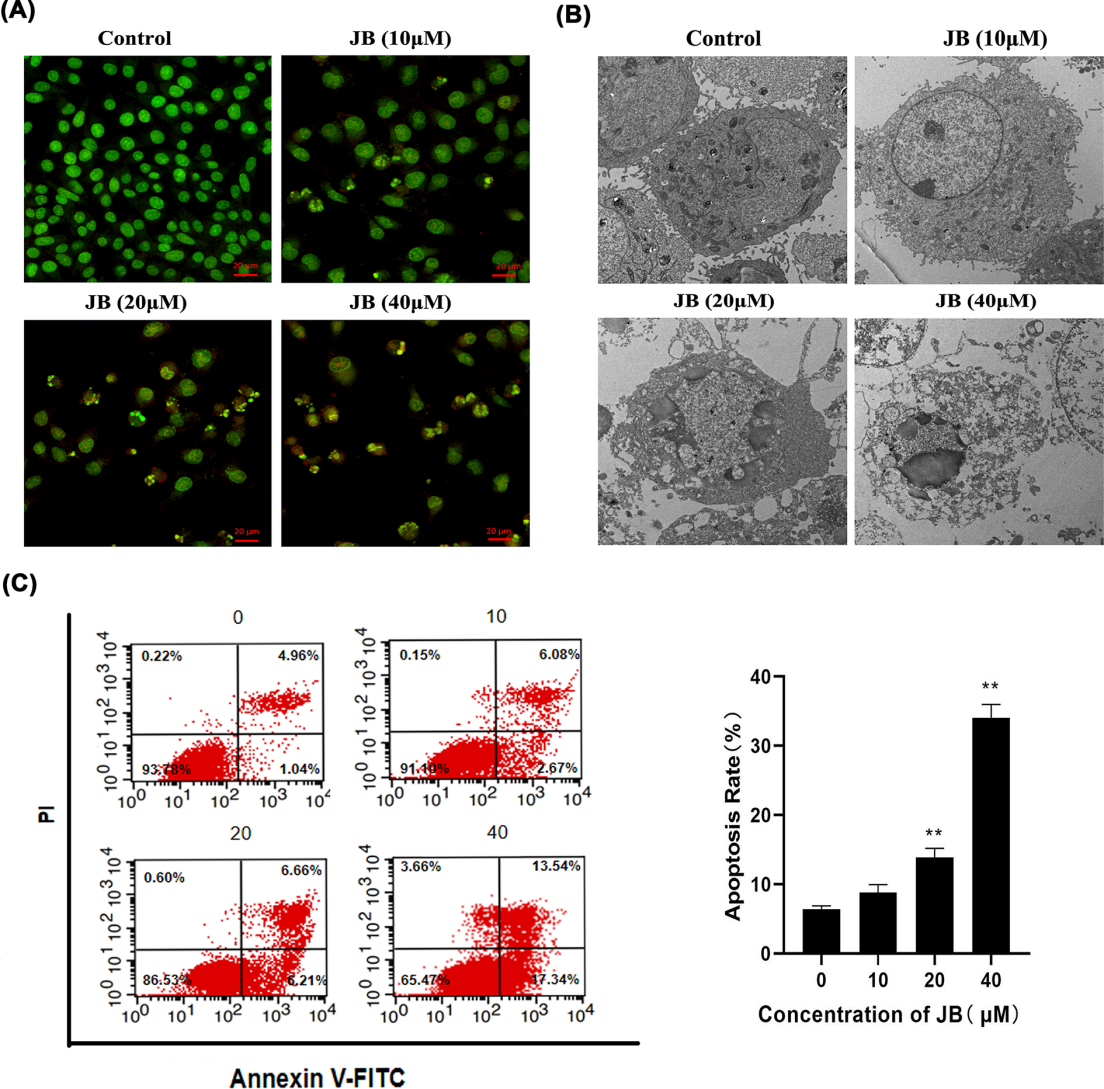
JB induced apoptosis of MKN45 cells MKN45 cells were treated with or without the indicated concentrations of JB for 24 h. (**A**) Cells stained with AO/EB and viewed under fluorescence microscopy showing apoptosis, Scale bar: 20 µm. (**B**) The ultrastructure of MKN45 cells were observed by transmission electron microscopy (×13K). (**C**) Apoptotic rates were quantified using an Annexin V-FITC/PI double staining cytometry. Data are expressed as means ± SD of three independent experiments. *P<0.05, **P<0.01 compared with the control group.

**Figure 5:**
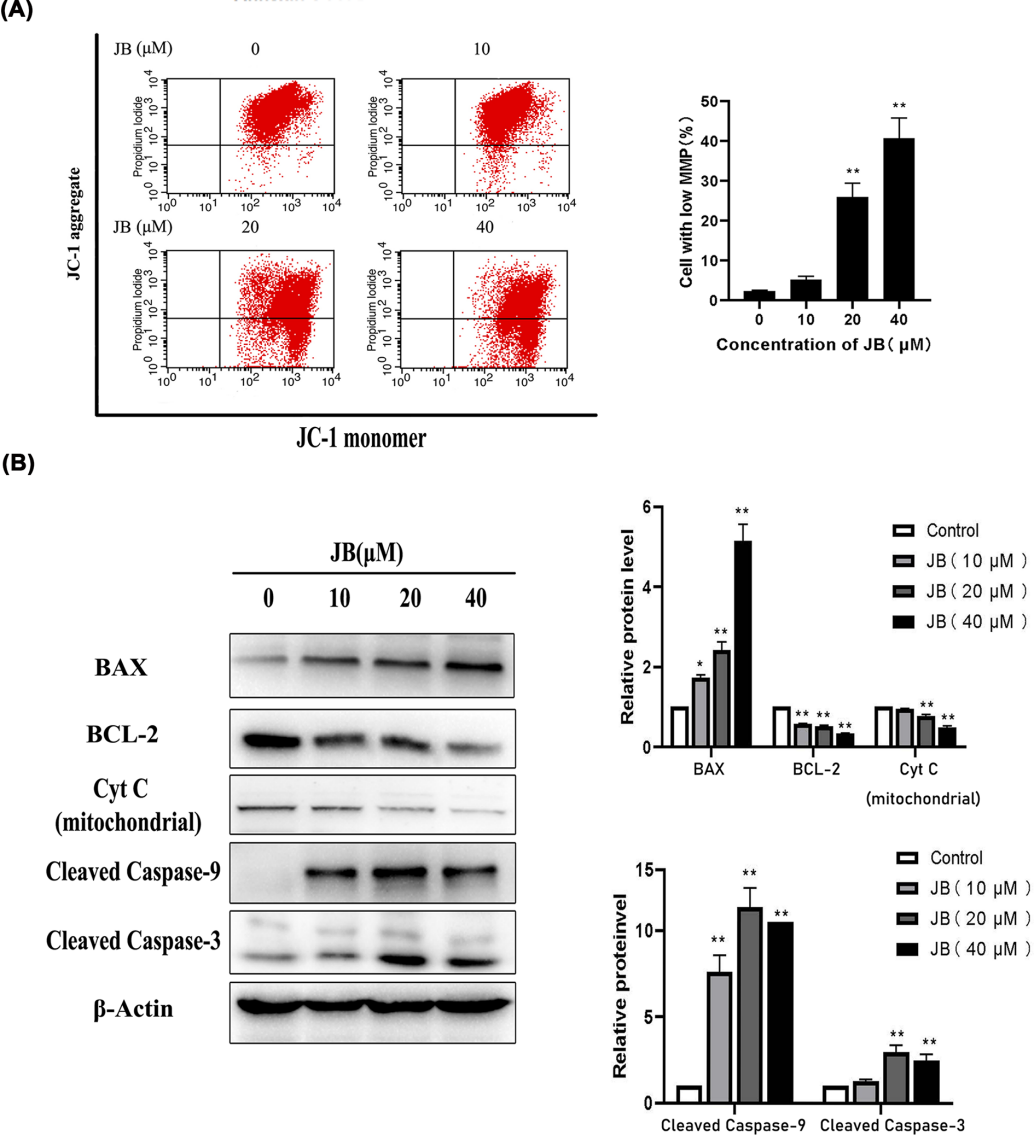
JB activated the mitochondrial apoptosis pathway MKN45 cells were treated with or without the indicated concentrations of JB for 24 h. (**A**) The mitochondrial membrane potential disruption by JB in MKN45 cells measured by flow cytometry (JC-1 staining). (**B**) Expression levels of apoptosis-related proteins including BAX, BCL-2, Cytochrome c (mitochondrial), Cleaved Caspase-9, Cleaved Caspase-3 were detected by western blotting. Data are expressed as means ± SD of three independent experiments. *P<0.05, **P<0.01 compared with the control group.

Morphological changes in MKN45 cells treated with JB for 24 h were observed under a laser confocal microscope after AO/EB staining. The normal cell nucleus presented a green fluorescence, while the early apoptotic cell nucleus showed a brighter green fluorescence, the late apoptotic cell nucleus showed an orange fluorescence and the structure of the apoptotic cell nucleus became condensed. AO/EB staining indicated that JB induced apoptosis in MKN45 cells (Figure 4A). Then, we used a transmission electron microscope to further observe changes in the ultrastructure of MKN45 cells treated with JB. In the control group, the cells displayed complete cell membrane and nuclear membrane, uniform chromatin, and the structures of the mitochondria were clear. The JB groups revealed typical features of apoptosis including cell shrinkage, membrane deformation, chromatin condensation, fragmentation, and apoptotic body formation (Figure 4B). The JB-induced apoptosis of MKN45 cells was quantified by flow cytometry with Annexin V-FITC/PI staining assay. As shown in Figure 4C, early apoptosis and late apoptosis markedly increased as the concentration of JB increased. A decline in the mitochondrial membrane potential (ΔΨm) is a marked event during early cell apoptosis, so we assessed ΔΨm via the JC-1 assay, and we found a decrease in ΔΨm when MKN45 cells were treated with JB (Figure 5A). These data demonstrated that JB might induce MKN45 cells apoptosis through the mitochondrial pathway.

To investigate the underlying mechanism associated with JB-induced apoptosis of gastric cancer, we checked the expression of regulatory molecules associated with the mitochondrial apoptosis pathway. The results are shown in Figure 5B and Supplementary Figure S2. The expression of BCL-2 was visibly decreased, and the expression of BAX was increased compared with the control. Additionally, JB treatment-induced Cytochrome c release from mitochondria and up-regulated the expression of Cleaved Caspase-9 and -3 proteins. Taken together, our results suggested that JB induced apoptosis in gastric cancer cell MKN45 via the mitochondrial pathway.

### JB inhibited tumor growth in the xenograft mouse model

**Figure 6:**
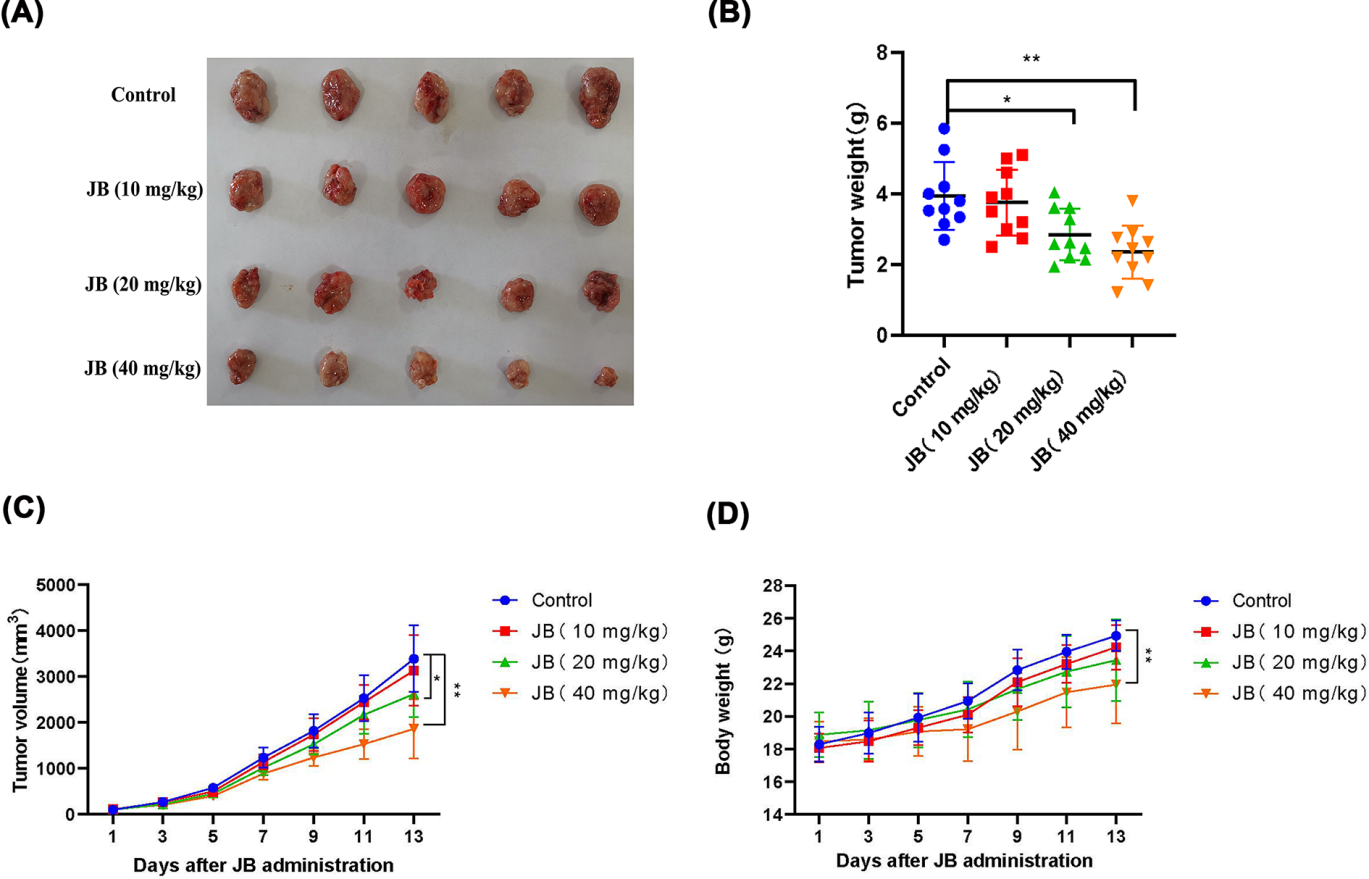
JB inhibited tumor growth in the xenograft mouse model MKN45 cells were implanted into the armpit of nude mice. Tumor-bearing mice were treated with JB (10, 20, and 40 mg/kg per day) for 2 weeks. (**A**) The photograph of the dissected tumors. (**B**) Tumor weights measured after tumor dissection. (**C**) Tumor volume was recorded during the treatment and growth curves were constructed. (**D**) Body weight was measured during the treatment and plotted. Data are expressed as means ± SD (*n*=10). *P<0.05, **P<0.01 compared with the control group.

To further confirm the antitumor effect of JB *in vivo*, we transplanted MKN45 cells into nude mice to study whether JB could suppress tumor growth *in vivo* (Figure 6A). The tumor weight (Figure 6B) and volume (Figure 6C) were greatly decreased in mice treated with JB medium- and high-dose group (20 and 40 mg/kg) compared with the control group. However, no significant difference was observed between mice treated with the low-dose group and the control group. In addition, all of the nude mice bearing tumors survived during the treatment. Compared with the control, the body weight significantly decreased in the high-dose group; however, there was no significant difference in the low- and medium-dose groups (Figure 6D).

## Discussion

*E. fischeriana* Steud has been used as a Chinese medicine remedy for cancer [5]. According to the literature, diterpenoids are the major components of *E. fischeriana* Steud [7–8]. However, little research has been reported on anticancer effects and mechanisms of the diterpenoids. JB is an ent-abietane-type diterpenoid from the plant. In the present study, we revealed JB inhibited the growth of MKN45 cells *in vitro* and suppressed tumor growth in nude mice *in vivo*. Subsequently, we investigated the anticancer molecular mechanism of JB.

The unlimited proliferation is the most important biological characteristic of malignant tumor cells [13]. JB was found to inhibit the clone formation and DNA biosynthesis of MKN45 cells. These results suggested that JB can interfere with cell proliferation. Cell proliferation is achieved through the cell cycle. Rapid cell cycle progression is one of the hallmarks of cancer [14]. Therefore, targeting the cell cycle has been considered an anticancer therapeutic strategy [15]. We observed that JB arrested the cell cycle at the S phase. The cell cycle is controlled by cyclins and cyclin-dependent protein kinases (CDKs) [16]. Different cyclin proteins are expressed at different stages of the cell cycle and bind to the corresponding CDKs to form Cyclin-CDK complexes that promote cell cycle progression. Cyclin A-CDK2 plays an important role in the regulation of S to G2 phase transition [17]. CDC25A has been shown to induce activation of Cyclin A-CDK2 complexes [18]. We found JB down-regulated CDK2, Cyclin A, and CDC25A protein levels. The above results suggested that JB inhibited cell proliferation by inducing S arrest in gastric cancer cells.

However, the molecular mechanism underlying JB-induced S-phase arrest in MKN45 cells remains poorly understood. DNA is the carrier of genetic information in nearly all living things. Numerous anticancer drugs induce DNA damage and checkpoint kinases, which cause cell cycle arrest, such as cisplatin, and camptothecin [19–22]. We speculated that JB might induce DNA damage in MKN45 cells. γ-H2AX can be used as a biomarker for DNA damage. Hence, we detected the levels of the γ-H2AX in JB-treated MKN45 cells by immunofluorescent staining and western blot analysis. We observed an increase in expression of γH2AX in MKN45 cells after JB treatment. These results indicated that JB induced massive DNA damage. ATR-CHK1 DNA damage response pathway plays a significant role in the regulation of S-phase arrest [23,24]. CHK1 is known to regulate the stability of CDC25A [25–27]. Based on the results of our study, we conjectured that the ATR-CHK1 pathway may be involved in JB-induced S-phase arrest. Thus, we first monitored the activation status of ATR and CHK1 after JB treatment by western blot analysis. Results showed that JB induced phosphorylation of ATR and CHK1 at the Thr1989 and Ser 345 site, respectively. Thus, these data demonstrated that JB induced S-phase cell cycle arrest in MKN45 cells by activating the ATR-CHK1 DNA damage checkpoint pathway.

Evading apoptosis is a hallmark of cancer. Induction of apoptosis is a strategy of anticancer therapy [28]. We observed that MKN45 cells treated with JB displayed the classic features of apoptosis such as cell shrinkage, the partition of cytoplasm, chromosome condensation, etc. Meanwhile, the apoptosis rate increased significantly. We continued to study the mechanism of JB-induced apoptosis. Mitochondrial apoptotic pathway is a classical intrinsic apoptotic pathway [29], BAX and BCL-2 are important regulatory proteins on this pathway [30]. Antiapoptotic protein BCL-2 binds to mitochondria, prevent the release of Cytochrome c from mitochondria into the cytoplasm [31]. Conversely, proapoptotic protein BAX is translocated to the mitochondrion and increases mitochondrial membrane permeability, causing a decrease in ΔΨm and Cytochrome c release, which stimulates caspase cascade-initiating apoptosis. We then examined whether mitochondrial apoptotic pathway was involved in JB-induced MKN45 cell apoptosis. Our experimental results showed that JB triggered MMP depolarization, facilitated Cytochrome c release from mitochondria, down-regulated expression of BCL-2 protein, and up-regulated BAX, Cleaved Caspase-9 and Cleaved Caspase-3 proteins expression in MKN45 cells. Our data indicated that JB induced MKN45 cells apoptosis through the mitochondrial apoptosis pathway.

## Conclusions

**Figure 7:**
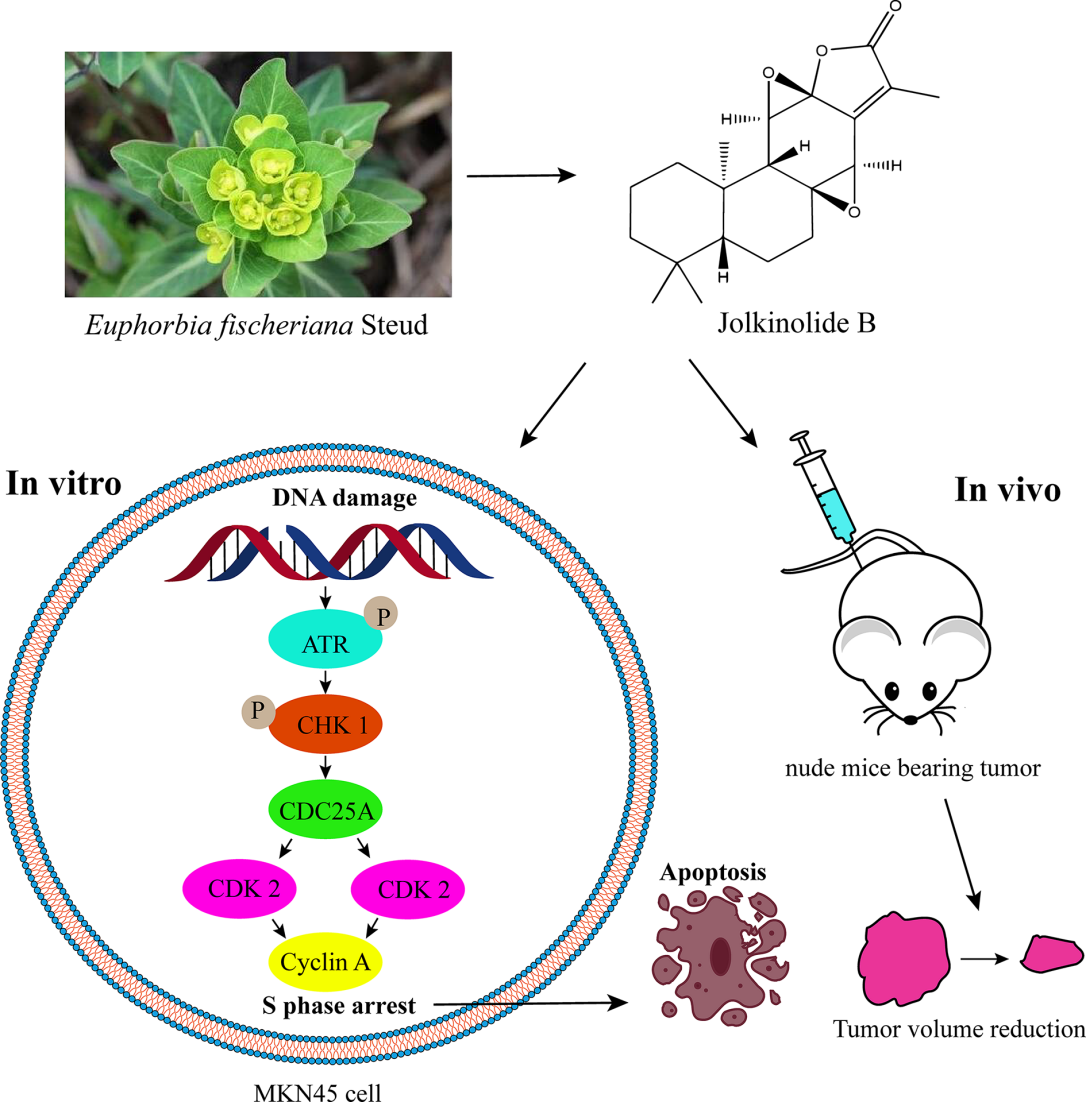
Schematic diagram depicting the anticancer mechanism of JB in MKN45 cells

JB inhibits not only the proliferation of gastric cancer cell MKN45 but also gastric xenograft tumor growth, which has an antigastric cancer effect *in vivo* and *in vitro*. Furthermore, the present study reveals its anticancer mechanism. On the one hand, JB causes DNA damage in gastric cancer MKN45 cells and induces the S cycle arrest by activating the ATR-CHK1-CDC25A-Cdk2 signaling pathway, On the other hand, JB induces MKN45 cells apoptosis through the mitochondrial pathway, and ultimately effectively inhibits the growth of gastric cancer cells. Our research shows that JB may be a potential antitumor agent against gastric carcinoma (Figure 7). In the follow-up experiment, we will further investigate the effect of JB on normal gastric cells and systematically evaluate the safety of JB.

## Supplementary Material

Supplementary Figures S1-S2 and Table S1

## Data Availability

The data that support the findings of the present study are available from the corresponding authors upon reasonable request.

## Abbreviations

AO/EB: acridine orange/ethidium bromide
BSA: bovine serum albumin
CDK: cyclin-dependent protein kinase
DAPI: 4′,6-diamidino-2-phenylindole
DSB: double-strand breaks
ECL: electrochemiluminescence
EdU: 5-ethynyl-2′-deoxyuridine
FITC: fluorescein isothiocyanate
IC50: 50% inhibitive concentration
JB: Jolkinolide B
MTT: 3-(4,5-dimethyl-2-thiazolyl)-2,5-diphenyl tetrazolium bromide
PBS: phosphate buffered saline
PI: propidium Iodide
PVDF: polyvinylidene difluoride
RIPA: radioimmunoprecipitation
SD: standard deviation
SDS-PAGE: sodium dodecyl sulfate polyacrylamide gel electrophoresis
TBS: tris-buffered saline
TBST: 1X tris-buffered saline with 0.05% tween 20
TEM: transmission electron microscope
V: tumor volume
