# Supplementary material for: Jolkinolide B induces cell cycle arrest and apoptosis in MKN45 gastric cancer cells and inhibits xenograft tumor growth in vivo
Source: Biosci Rep. 2022 Jun 27;42(6):BSR20220341. doi: 10.1042/BSR20220341 (PMC9245080; doi:10.1042/BSR20220341)
Supplement: Supplementary Figures S1-S2 and Table S1 [file BSR-2022-0341_supp.pdf]

Jolkinolide B from *Euphorbia fischeriana* Steud Induces Cell Cycle Arrest and Apoptosis in MKN45 Gastric Cancer Cells and Inhibits Xenograft Tumor Growth In Vivo

Hao Zhang[1], Jiayi Qian[2], Ming Jin[1], Li Fan[1], SongJie Fan[1], Hong Pan[2], Yang Li[1], Ningning Wang[1], Baiyu Jian[1]*

1. Supplementary Table

Supplementary Table S1. Information on primary and secondary antibodies used in the western blot analysis of this study.

primary Antibody	Catalogue	Secondary antibody	Provider	Dilution
anti-ATR	13934	Rabbit	Cell Signaling	1:1000
anti-p-ATR	30632	Rabbit	Cell Signaling	1:1000
anti-Chk1	2360	Mouse	Cell Signaling	1:1000
anti-p-Chk1	2348	Rabbit	Cell Signaling	1:1000
anti Cdc25A	3652	Rabbit	Cell Signaling	1:1000
anti-CDK2	18048	Rabbit	Cell Signaling	1:1000
anti-Cyclin A2	4656	Mouse	Cell Signaling	1:1000
anti-γ-H2AX	9718	Rabbit	Cell Signaling	1:1000
anti-Cytochrome C	11940	Rabbit	Cell Signaling	1:1000
anti-Cleaved Caspase-3	9661	Rabbit	Cell Signaling	1:500
anti-Cleaved Caspase-9	52873	Rabbit	Cell Signaling	1:1000
anti-Bax	5023	Rabbit	Cell Signaling	1:1000
anti-Bcl-2	15071	Mouse	Cell Signaling	1:1000
anti-β-actin	4970	Rabbit	Cell Signaling	1:1000

2. Supplementary Figures

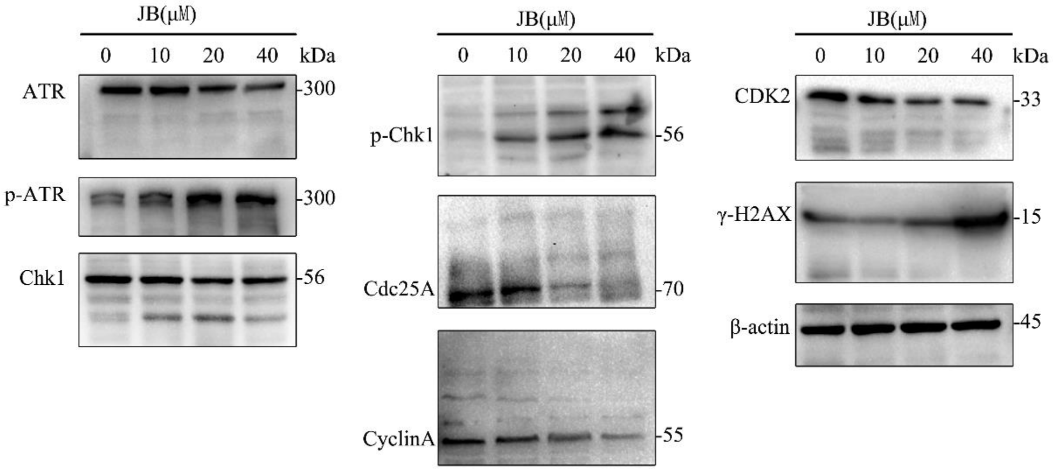

Supplementary Figure S1. Original, uncropped images of the western blots for Fig. 2C displayed in the text and results: The bands of ATR, p-ATR, Chk1, p-Chk1, Cdc25A, CyclinA, CDK2 ,r-H2AX and their expected molecular weight, with β-actin used as the protein loading control.

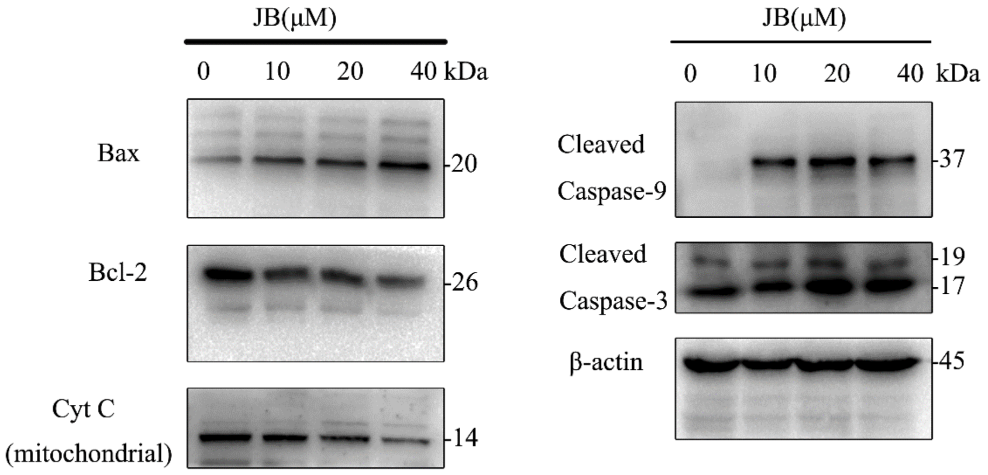

Supplementary Figure S2. Original, uncropped images of the western blots for Fig. 3E displayed in the text and results: The bands of Bax, Bcl-1, Cytochrome C (mitochondrial) , Cleaved Caspase-9, anti-Cleaved Caspase-3 and their expected molecular weight, with β-actin used as the protein loading control.
